# Ascorbate peroxidase 4 plays a role in the tolerance of *Chlamydomonas reinhardtii* to photo-oxidative stress

**DOI:** 10.1038/s41598-020-70247-z

**Published:** 2020-08-06

**Authors:** Eva YuHua Kuo, Meng-Siou Cai, Tse-Min Lee

**Affiliations:** 1 Department of Marine Biotechnology and Resources, National Sun Yat-Sen University, Kaohsiung, 80424 Taiwan; 2 Doctoral Degree Program in Marine Biotechnology, National Sun Yat-Sen University, Kaohsiung, 80424 Taiwan

**Keywords:** Plant physiology, Plant stress responses

## Abstract

Ascorbate peroxidase (APX; EC 1.11.1.11) activity and transcript levels of *CrAPX1*, *CrAPX2*, and *CrAPX4* of *Chlamydomonas reinhardtii* increased under 1,400 μE·m^−2^·s^−1^ condition (HL). CrAPX4 expression was the most significant. So, CrAPX4 was downregulated using amiRNA technology to examine the role of APX for HL acclimation. The *CrAPX4* knockdown amiRNA lines showed low APX activity and CrAPX4 transcript level without a change in CrAPX1 and CrAPX2 transcript levels, and monodehydroascorbate reductase (MDAR), dehydroascorbate reductase (DHAR), and glutathione reductase (GR) activities and transcript levels. Upon exposure to HL, *CrAPX4* knockdown amiRNA lines appeared a modification in the expression of genes encoding the enzymes in the ascorbate–glutathione cycle, including an increase in transcript level of CrVTC2, a key enzyme for ascorbate (AsA) biosynthesis but a decrease in MDAR and DHAR transcription and activity after 1 h, followed by increases in reactive oxygen species production and lipid peroxidation after 6 h and exhibited cell death after 9 h. Besides, AsA content and AsA/DHA (dehydroascorbate) ratio decreased in *CrAPX4* knockdown amiRNA lines after prolonged HL treatment. Thus, CrAPX4 induction together with its association with the modulation of MDAR and DHAR expression for AsA regeneration is critical for *Chlamydomonas* to cope with photo-oxidative stress.

## Introduction

Reactive oxygen species (ROS) are generated in plants upon exposure to stressful conditions^1^. Upon high intensity illumination, the photosynthetic electron transport components will be over-reduced, and O_2_ will be photoreduced via photosystem I and photosystem II for formation of ROS^2^, which oxidize macromolecules (lipids, proteins, and nucleic acids) and subsequently impact cellular metabolism and physiological performance^3^. To counter the ROS-induced oxidative stress, plants have developed the antioxidative defense system encompassing antioxidants and antioxidative enzymes, such as ascorbate (AsA) and ascorbate peroxidase (APX; EC 1.11.1.11). APX, which is the first step of the AsA-glutathione (GSH) cycle, uses AsA as its specific electron donor to reduce H_2_O_2_ to water.

APX, a central enzyme for ROS scavenging in plants^4,5^, can be induced under abiotic and biotic stresses^6–11^. Salt stress can increase *OsAPX2* and *OsAPX7* transcript levels but decreases *OsAPX8* transcript level in rice^12^ while drought stress also increases APX transcript level in rice^13^ and wheat^14^. Transcript levels of APXs in potato tubers^15^, rice^7^, and *Arabidopsis*^11^ are also induced by low temperature exposure. The cytosolic APX (APX1 and APX2) transcript level in *Arabidopsis thaliana* also increases by excess light illumination at 2,000 μE·m^−2^·s^−1^ within 15 min and reaches the maximum after 60 min, followed by a decrease^16^. This rapid increase is associated with the signal derived from a change in redox status of the plastoquinone pool caused by photoinhibition under high light condition. In spinach, the transcript level, protein level, and enzyme activity of cytosolic but not chloroplastic and mitochondrial APX increase when illuminated at 1,600 μE·m^−2^·s^−1^^10^. To date, few studies have focused on the effect of high-intensity illumination on the expression of APX in the green alga *Chlamydomonas reinhardtii*. In an investigation of the role of H_2_O_2_ as a signal in the activation of catalase in *C. reinhardtii* under 700 μE·m^−2^·s^−1^ illumination, Michelet et al. (2013)^17^ identify that accumulated H_2_O_2_ plays a role for high-light induction of APX transcription.

Previously, we identified that monodehydroascorbate reductase (MDAR)^18^, dehydroascorbate reductase (DHAR)^19^, and glutathione reductase (GR)^20^ are essential for *C*. *reinhardtii* against high light (HL) stress, but the role of APX in high light tolerance is not examined yet. Three APX isoforms are found in *Chlamydomonas* (Phytozome; https://www.phytozome.net/), *CrAPX1* (Cre02.g087700.t1.2), *CrAPX2* (Cre06.g285150.t1.2), and *CrAPX4* (Cre05.g233900.t1.2). Using the subcellular localization prediction programs, ChloroP 1.1 (https://www.cbs.dtu.dk/services/ChloroP/) and TargetP 1.1 (https://www.cbs.dtu.dk/services/TargetP/), we found that CrAPX1 and CrAPX2 show dual localization in chloroplast and mitochondrium and CrAPX4 is a chloroplastic enzyme (Supplementary Tab. S1). During plant evolution, more APX isoforms with different localization appear in vascular plants. For example, three cytosolic (APX1, APX2, APX6), two chloroplastic (stromal sAPX, thylakoid tAPX), and three peroxisomal (APX3, APX4, APX5) isoforms are detected in Arabidopsis^21–24^. Later, the evolution analysis of APX isoforms in the chloroplast by Maruta, Sawa, Shigeoka and Ishikawa (2016)^25^ shows a similar result in Arabidopsis but APX4 and APX6 are excluded because they do not have APX activity due to the substitution of nucleotides or amino acids essential for APX activity. They also find that only one chloroplastic APX protein, that is, CrAPX1, exists in *Chlamydomonas* and no APX isoforms can be detected in cytosolc and peroxisome. Further, the proteomic investigation of the *Chlamydomonas deg1c* mutant lacking DEG1C protease activity shows an increase of CrAPX4 protein in the chloroplast stroma^26^. It demonstrates that, in addition to CrAPX1, CrAPX4 also exists in *Chlamydomonas* chloroplast. Here, we found that the transcript levels of three *Chlamydomonas* APX isoforms increased under 1,400 μE·m^−2^·s^−1^ condition. Because CrAPX4 showed the most significant expression, CrAPX4 was downregulated via amiRNA-mediated knockdown using pChlamiRNA3 vector (Supplementary Fig. S1) to examine the role of APX in HL tolerance. Furthermore, the expression of MDAR, DHAR, and GR in *CrAPX4* knockdown amiRNA lines as well as ascorbate hemostasis was examined to see whether the ascorbate–glutathione cycle was affected by the knockdown of CrAPX4 expression.

## Results

### Growth, oxidative response, and APX expression to HL stress

**Figure 1:**
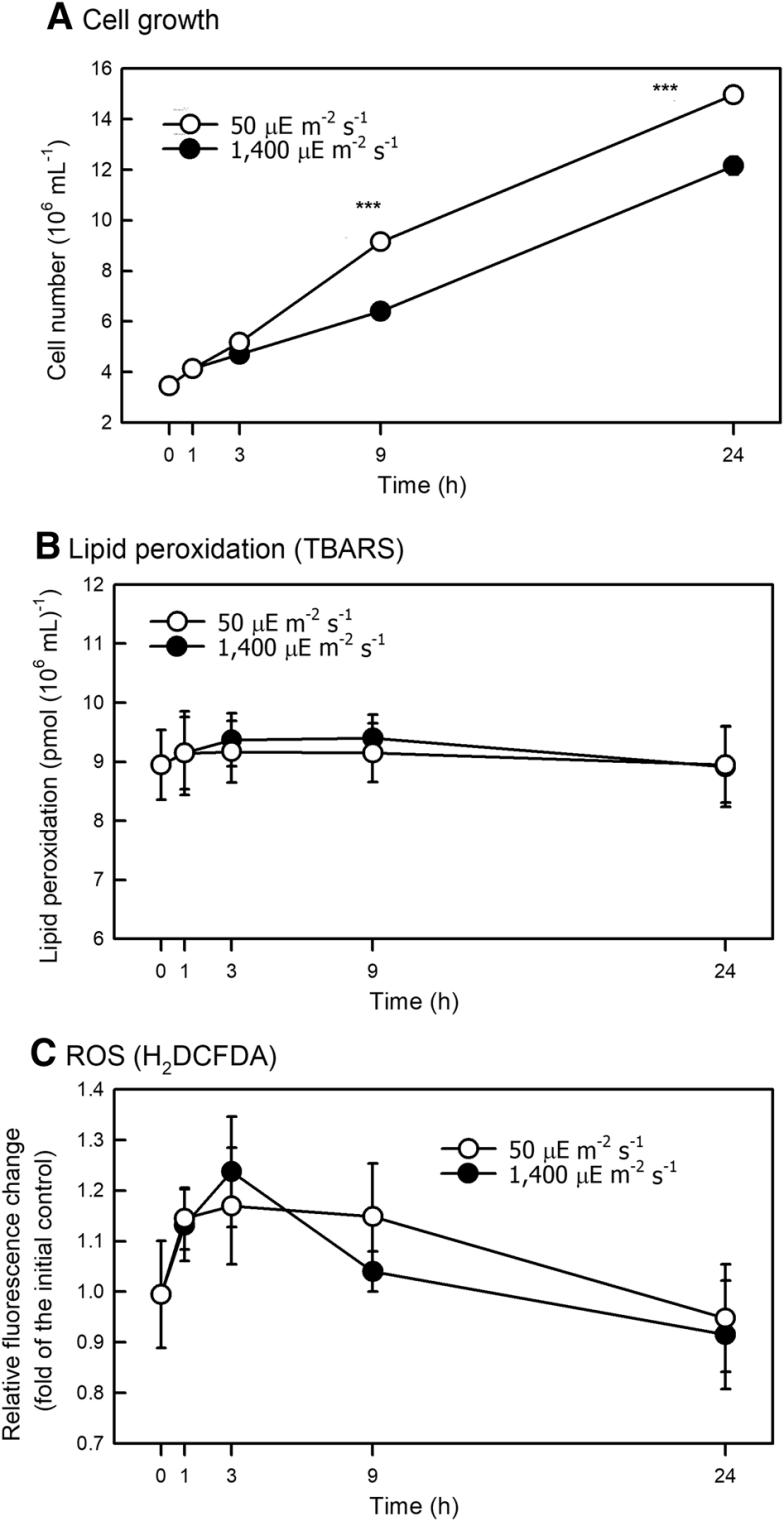
Changes in cell growth (**A**), lipid peroxidation (TBARS) (**B**), and ROS (H_2_DCFDA) (C) in *Chlamydomonas reinhardtii* CC-400 under 50 (NL) and 1,400 (HL) μE·m^−2^·s^−1^ conditions. Data are expressed as the mean ± SD (n = 3) and analyzed by the *t*-test.

The growth ability of HL-treated cells slightly decreased compared to NL condition (Fig. 1A), while lipid peroxidation (TBARS) (Fig. 1B) and ROS production (H_2_DCFDA) (Fig. 1C) were not increased.

**Figure 2:**
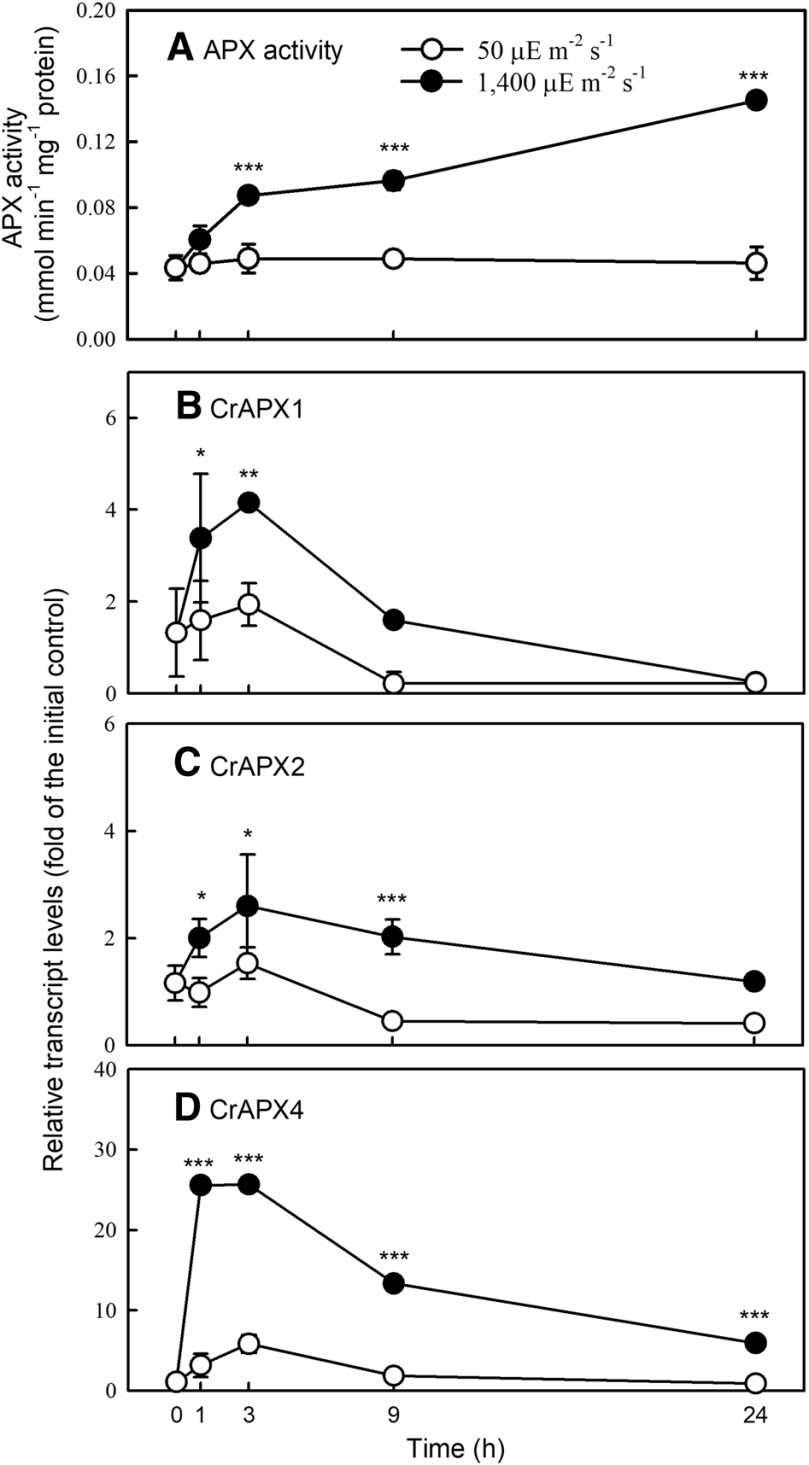
Changes in APX activity (**A**) and transcript levels of *CrAPX1* (**B**), *CrAPX2* (**B**), and *CrAPX4* (**B**) in *Chlamydomonas reinhardtii* under 50 (NL) and 1,400 (HL) μE·m^−2^·s^−1^ conditions. Data are expressed as the mean ± SD (n = 3). The asterisk indicates a significant difference between NL and HL treatments at the same time point by the *t*-test (*P < 0.05; **P < 0.01; ***P < 0.001).

APX activity increased 3 h after HL treatment (Fig. 2A) while transcript levels of *CrAPX1* (Fig. 2B), *CrAPX2* (Fig. 2C), and *CrAPX4* (Fig. 2D) also increased with a peak around 1–3 h. Among these isoforms, *CrAPX4* showed a 25-fold increase in transcript level while *CrAPX1* and *CrAPX2* transcript levels exhibited 4- and 2-fold increase, respectively.

### Selection of *CrAPX4* downregulation lines

**Figure 3:**
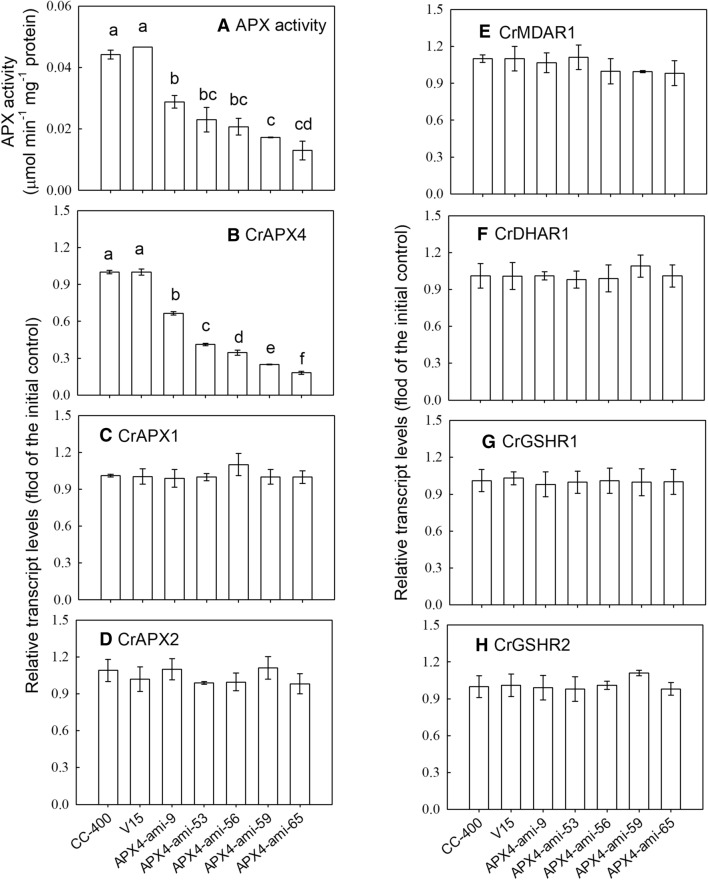
APX activity (**A**) and transcript levels of CrAPX4 (**B**), CrAPX1 (**C**), CrAPX2 (**D**), CrMDAR1 (**E**), CrDHAR1 (**F**), CrGSHR1 (**G**), and CrGSHR2 (**H**) in *Chlamydomonas reinhardtii* wild-type CC-400, vector-only control (V15), and *CrAPX4* knockdown lines (APX4-ami 9, APX4-ami 53, APX4-ami 56, APX4-ami 59, and APX4-ami 65) under 50 μE·m^−2^·s^−1^ condition. The data are expressed as the mean ± SD (n = 3) and different symbols indicate significant difference analyzed by Duncan’s new multiple range test (*P* < 0.05).

First, *CrAPX4*-knockdown amiRNA lines were screened by examination of their APX activity. Five *CrAPX4*-knockdown amiRNA lines, APX4-ami-9, APX4-ami-53, APX4-ami-56, APX4-ami-59, and APX4-ami-65, displaying low APX activity compared with the wild type and the APX activity of the vector-only line (V15) was equivalent to that of wild type (Fig. 3A). The transcript levels of these *CrAPX4*-knockdown amiRNA lines were also depressed while the vector-only line (V15) showed the same *CrAPX4* transcript level as the wild-type (Fig. 3B). Whether the downregulation of CrAPX4 expression affected the expression of other APX isoforms and the genes encoding the enzymes in the ascorbate–glutathione cycle was elucidated. The transcript levels of CrAPX1 (Fig. 3C) and CrAPX2 (Fig. 3D) as well as transcript levels of CrMDAR1 (Fig. 3E), CrDHAR1 (Fig. 3F), CrGSHR1 (Fig. 3G), and CrGSHR2 (Fig. 3H) in these *CrAPX4*-knockdown amiRNA lines were similar as those of wild type and vector-only control. The activities of these enzymes in the *CrAPX4*-knockdown amiRNA lines were not affected as compared to the wild type and vector-only control under NL condition (data not shown).

### Increased sensitivity to HL stress by *CrAPX4* downregulation

**Figure 4:**
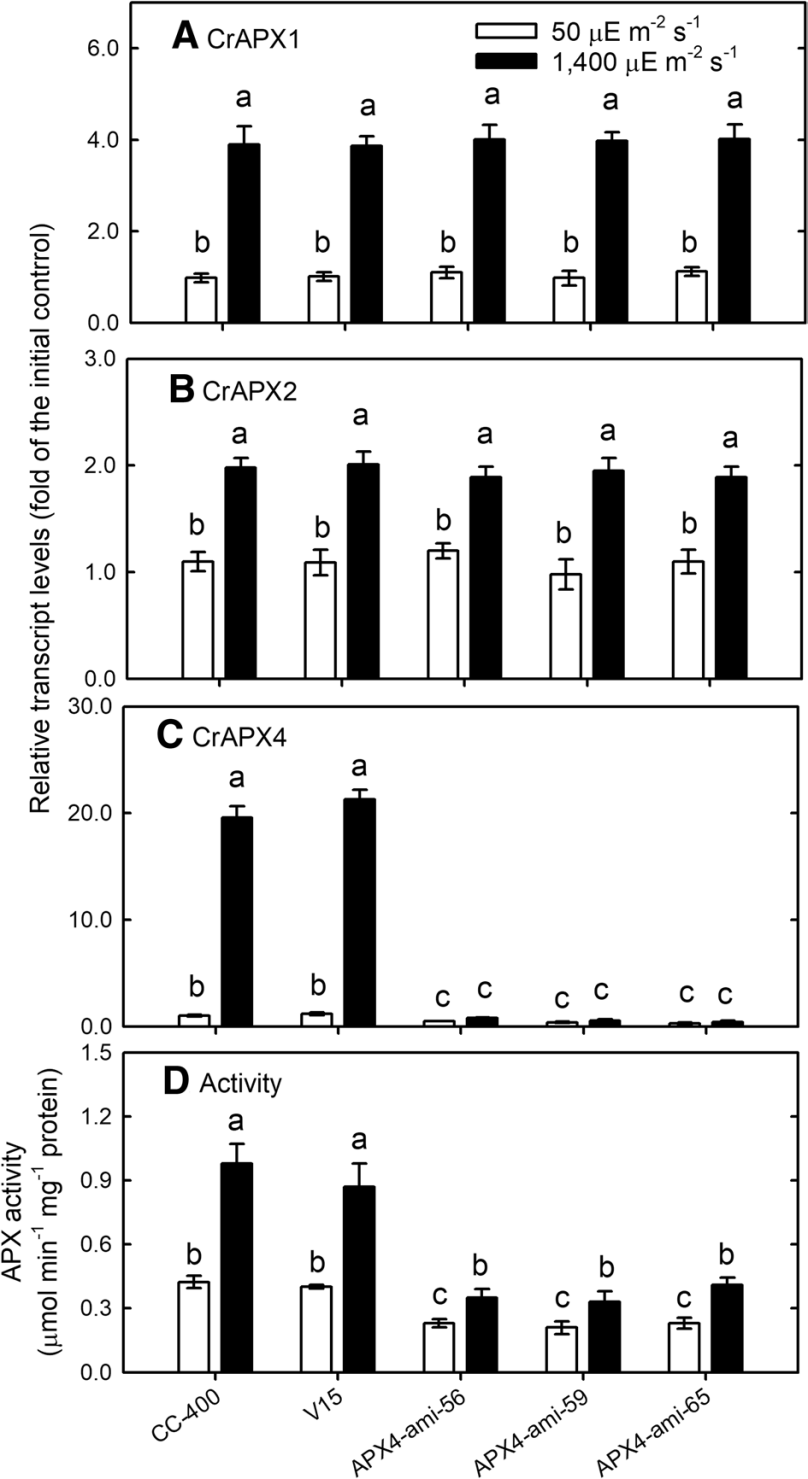
The transcript levels of CrAPX1 (**A**), CrAPX2 (**B**), and CrAPX4 (**C**) and the activity of APX (**D**) in *Chlamydomonas reinhardtii* wild-type CC-400, vector-only control (V15), and *CrAPX4* knockdown lines (APX4-ami 56, APX4-ami 59, and APX4-ami 65) under 50 μE·m^−2^·s^−1^ condition. The data are expressed as the mean ± SD (n = 3) and different symbols indicate significant difference analyzed by Duncan’s new multiple range test (*P* < 0.05).

Because the APX activity in the APX4-ami-9 line showed a less decline and the APX4-ami-53 line was contaminated, they were not used further. Transcript levels of CrAPX1 (Fig. 4A) and CrAPX2 (Fig. 4B) of APX4-ami-56, APX4-ami-59, and APX4-ami-65 lines increased under HL condition, which were same as those of wild type and vector-only control. CrAPX4 transcript levels of *CrAPX4*-knockdown amiRNA lines did not increase under HL condition while those of wild-type and vector-only control showed a significant increase (Fig. 4C). The APX activity in wild type ad vector-only lines increased under HL condition but that in APX4-ami-56, APX4-ami-59, and APX4-ami-65 lines did not increase (Fig. 4D). The cells of these *CrAPX4*-knockdown amiRNA lines remained green till 6 h after HL treatment but bleached after prolonged HL treatment (9 h) (Supplementary Fig. S2).

**Figure 5:**
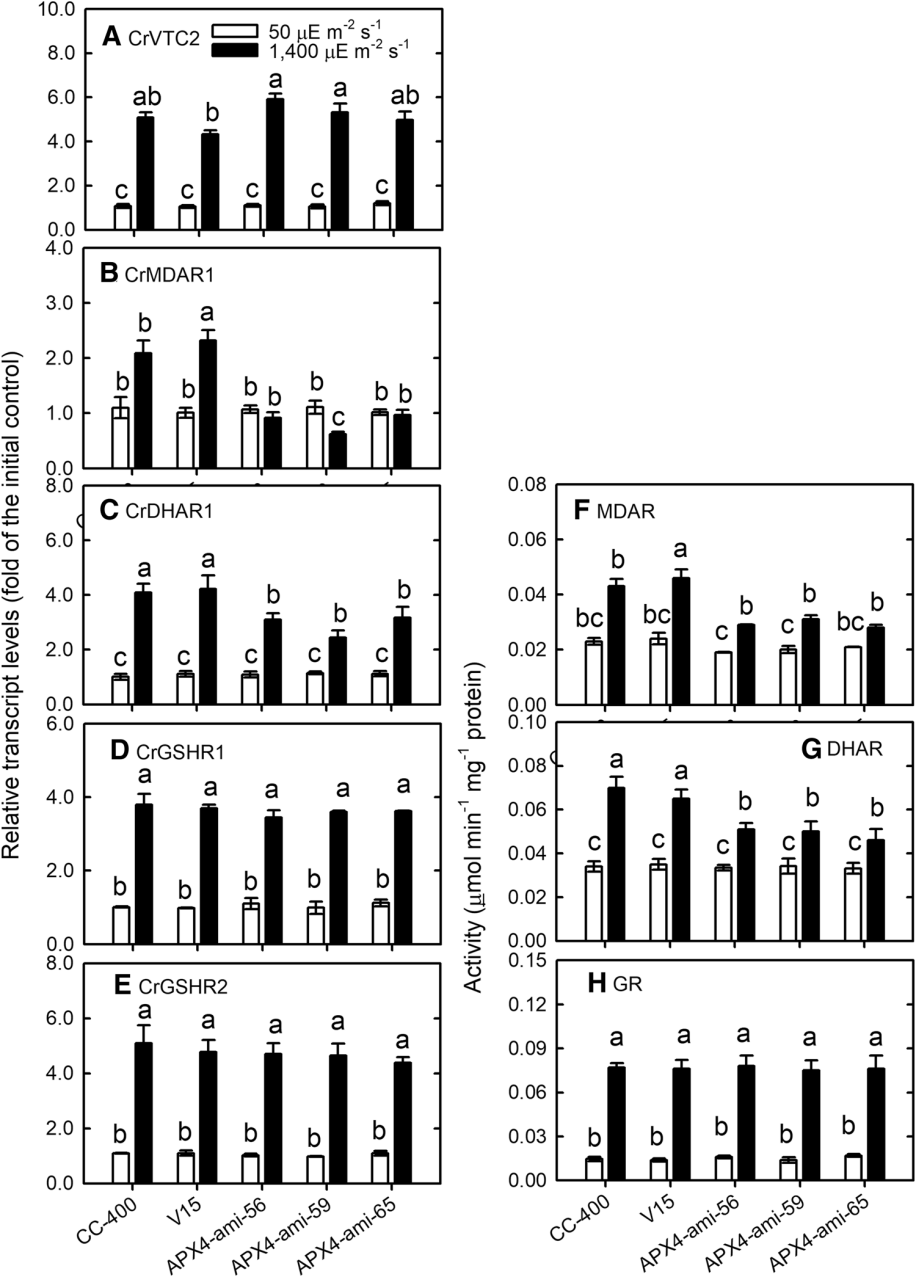
The changes in the expression of genes and activity of enzymes in the ascorbate–glutathione cycle under HL condition. The transcript levels of CrVTC2 (**A**), CrMDAR1 (**B**), CrDHAR1 (**C**), CrGSHR1 (**D**) and CrGSHR2 (**E**) and the activity of MDAR (**F**), DHAR (**G**), and GR (**H**) in *Chlamydomonas reinhardtii* wild-type CC-400, vector-only control (V15), and *CrAPX4* knockdown lines (APX4-ami 56, APX4-ami 59, and APX4-ami 65) 1 h after exposure to 50 (NL) and 1,400 (HL) μE·m^−2^·s^−1^. The data are expressed as the mean ± SD (n = 3) and different symbols indicate significant difference analyzed by Duncan’s new multiple range test (*P* < 0.05).

Whether CrAPX4 downregulation can modify the expression of CrVTC2, a key enzyme for ascorbate synthesis in *C*. *reinhardtii*^27^, and other enzymes in the ascorbate–glutathione cycle was evaluated. Transcript level of CrVTC2 was same in wild type, vector-only control, and *CrAPX4*-knockdown amiRNA lines under NL condition, and showed a similar increase under HL condition (Fig. 5A). CrMDAR1 (Fig. 5B) and CrDHAR1 (Fig. 5C) transcript levels in wild type and vector-only control increased under HL condition, but CrMDAR1 transcript level in *CrAPX4*-knockdown amiRNA lines did not increase while CrDHAR1 transcript level slightly increased. For GR genes, transcript levels of CrGSHR1 (Fig. 5D) and CrGSHR2 (Fig. 5E) showed a similar increase in wild type, vector-only control, and *CrAPX4*-knockdown amiRNA lines under HL condition.

HL illumination increased MDAR (Fig. 5F) and DHAR (Fig. 5G) activities but a less increase for *CrAPX4*-knockdown amiRNA lines, while GR activity showed a similar increase in wild type, vector-only control, and *CrAPX4*-knockdown amiRNA lines (Fig. 5H).

**Figure 6:**
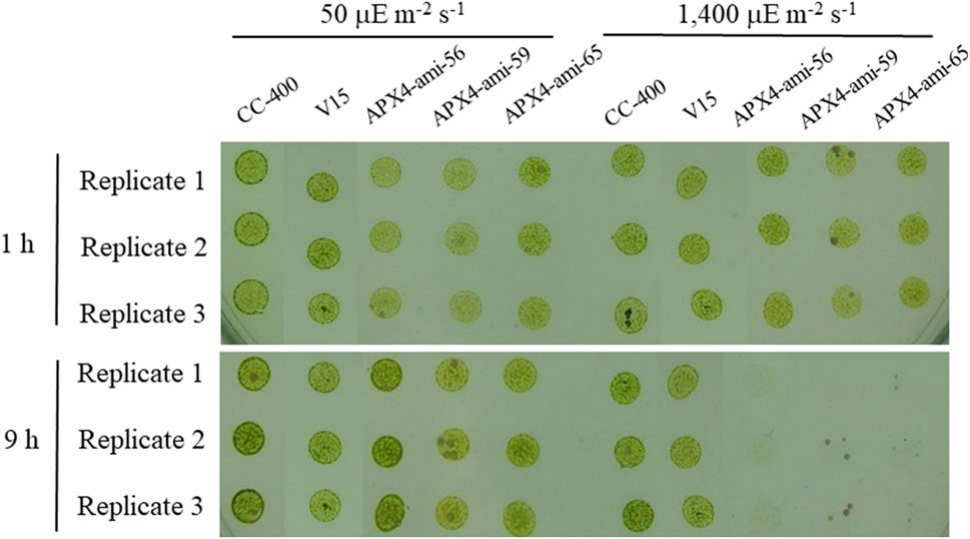
The viability assay of *Chlamydomonas reinhardtii* wild-type CC-400, vector-only control (V15), and *CrAPX4* knockdown lines (APX4-ami 56, APX4-ami 59, and APX4-ami 65) 1 h (**A**) and 9 h (**B**) after exposure to 50 (NL) and 1,400 (HL) μE·m^−2^·s^−1^. Three biological replicates have been shown and the cell colony in the *CrAPX4* knockdown lines was normal 1 h after HL treatment but absent after prolonged HL treatment (9 h).

The viability assay showed that APX4-ami-56, APX4-ami-59, and APX4-ami-65 lines died 9 h after HL treatment while wild type and vector only control exhibited a normal growth ability (Fig. 6).

**Figure 7:**
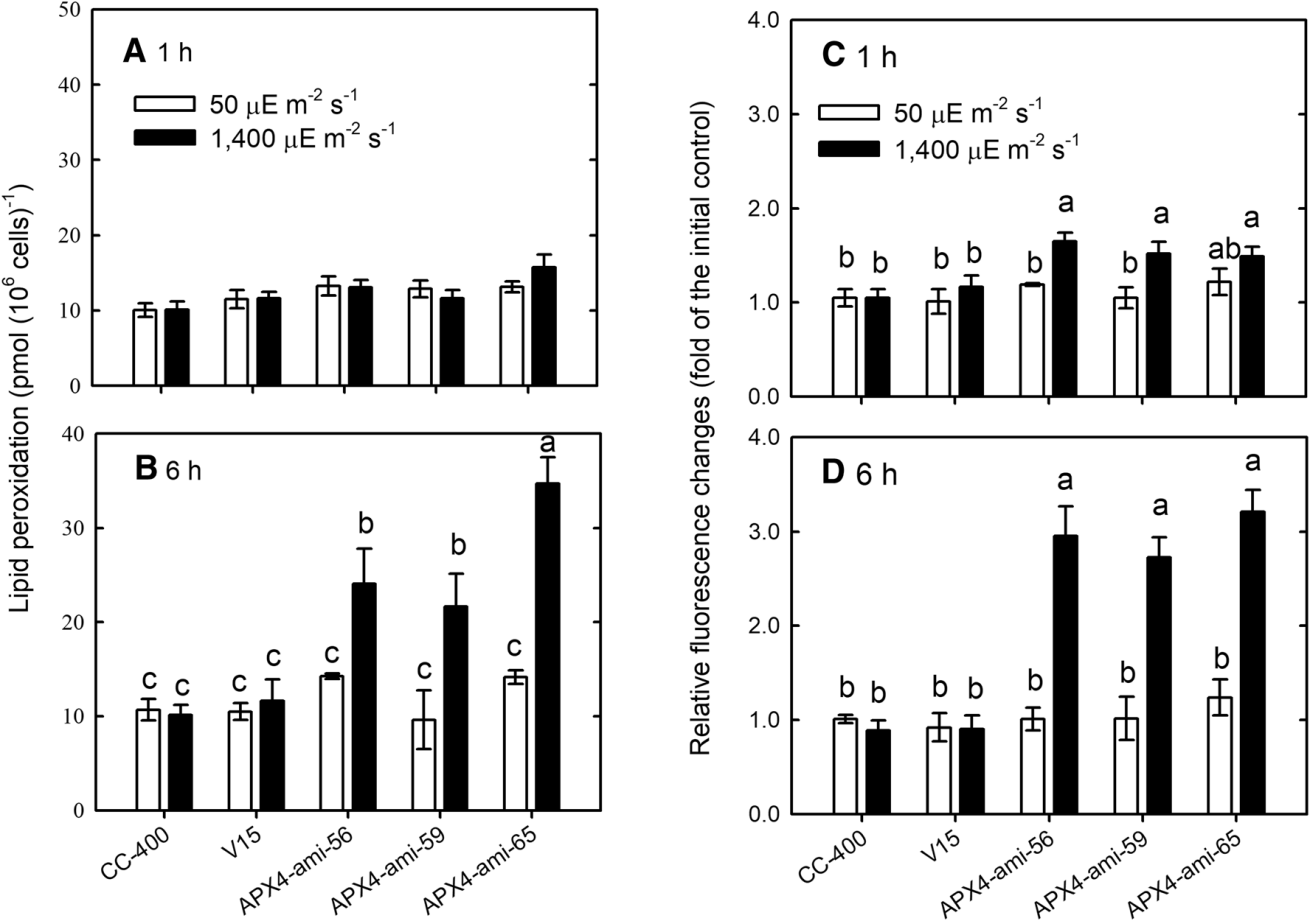
Lipid peroxidation (TBARS) (**A**, **B**) and ROS (H_2_DCFDA fluorescence) (**C**, **D**) of *Chlamydomonas reinhardtii CrAPX4* knockdown lines (APX4-ami 56, APX4-ami 59, and APX4-ami 65) under 50 (NL) and 1,400 (HL) μmol·m^−2^·s^−1^ conditions for 1 h (**A**, **C**) and 6 h (**B**, **D**). The controls are the wild-type CC-400 and vector-only line (V15). The data are expressed as the mean ± SD (n = 3) and different symbols indicate significant difference analyzed by Duncan’s new multiple range test (*P* < 0.05).

To estimate oxidative stress under HL condition, TBARS and ROS contents were determined. TBARS contents were not affected in wild-type and vector-only control under HL condition, and those in *CrAPX4* knockdown lines were also not affected after 1 h (Fig. 7A) but significantly increased after 6 h (Fig. 7B). Similarly, ROS content detected using 2′, 7′-dichlorodihydrofluorescein diacetate (H_2_DCFDA) in *CrAPX4* knockdown amiRNA lines slightly increased 1 h after HL treatment (Fig. 7C) and markedly increased after 6 h (Fig. 7D). Obviously, CrAPX4 downregulation elicited oxidative stress after prolonged HL treatment (6 h) and finally caused cell death after 9 h (Supplementary Fig. S2).

**Figure 8:**
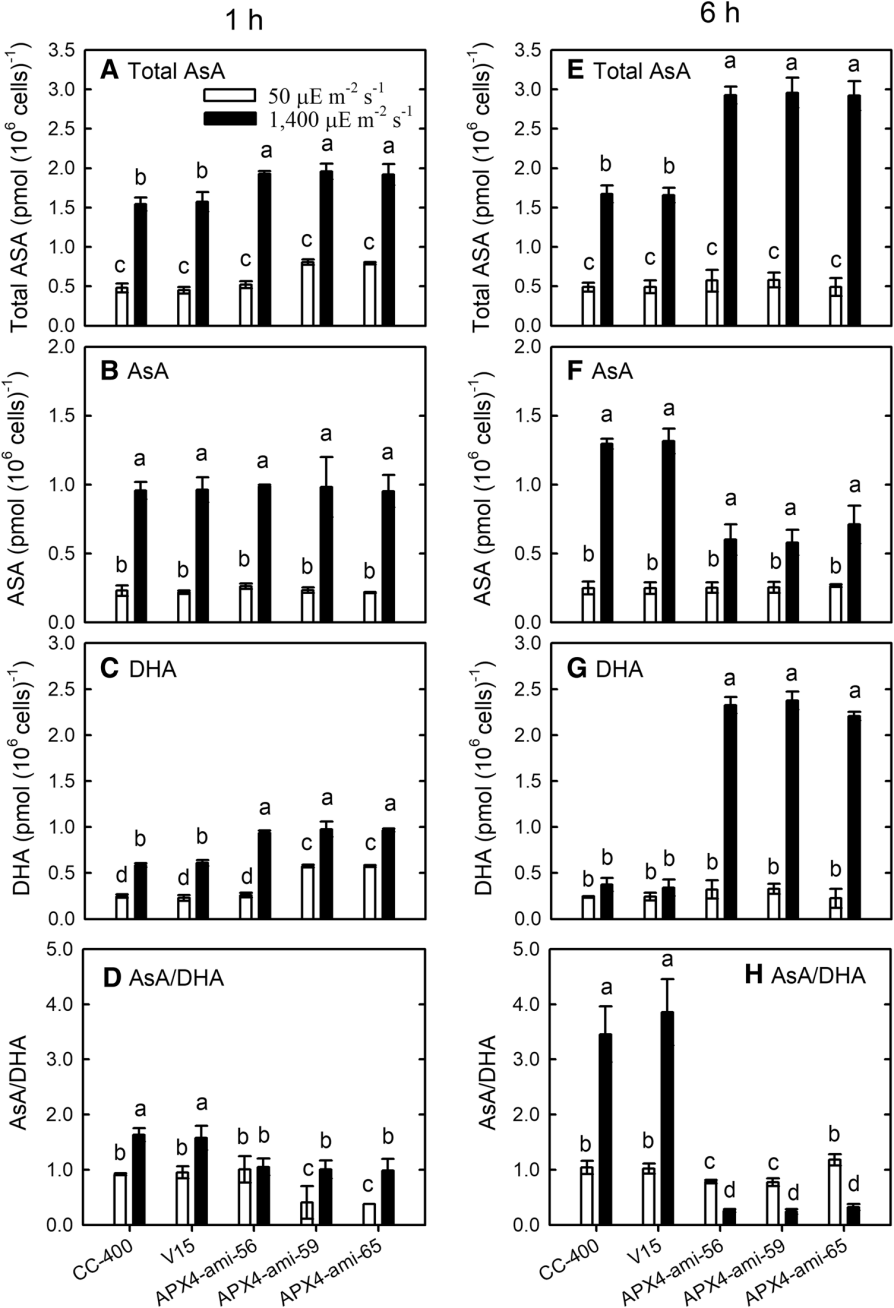
Changes in total AsA (**A**, **E**), AsA (**B**, **F**), DHA (**C**, **G**), and AsA/DHA ratio (**D**, **H**) of the *Chlamydomonas reinhardtii CrAPX4* knockdown lines (APX4-ami 56, APX4-ami 59, and APX4-ami 65) under 50 (NL) and 1,400 (HL) μE·m^−2^·s^−1^ conditions for 1 h and 6 h. The controls are the wild-type CC-400 and vector-only line (V15). Data are expressed as the mean ± SD (n = 3) and different symbols indicate significant difference by Duncan’s new multiple range teat (*P* < 0.05).

Under HL treatment for 1 h, total AsA (Fig. 8A) and DHA (Fig. 8C) contents exhibited a higher increase in *CrAPX4*-knockdown amiRNA lines than wild type and vector-only control, while AsA content (Fig. 8B) showed a similar increase in wild type, vector-only control, and *CrAPX4*-knockdown amiRNA lines. AsA/DHA ratio significantly increased in wild type and vector-only control under HL condition, but did not increase in APX4-ami-56 knockdown line and only slightly increased in APX4-ami-59 and APX4-ami-65 knockdown lines (Fig. 8D). As compared to 1-h HL treatment, total AsA content in *CrAPX4*-knockdown amiRNA lines showed a further increase after 6 h of HL treatment (Fig. 8E), whereas AsA content appeared a less increase than wild type and vector-only control (Fig. 8F) accompanied with a higher increase in DHA content (Fig. 8G) and a significant decline in AsA/DHA ratio (Fig. 8H). Ascorbate was not determined 9 h after HL treatment due to cell bleaching.

## Discussion

APX is crucial for *C*. *reinhardtii* to cope with photo-oxidative stress. Although algal cell growth slightly depressed under HL condition, no oxidative stress occurred. In wild type, an increase in expression of APX together with MDAR, DHAR, and GR under HL condition demonstrates that ROS over-accumulation and oxidative stress can be effectively prevented via the ascorbate–glutathione cycle. We have previously identified that MDAR^18^, DHAR^19^, and GR^20^ are essential for *C*. *reinhardtii* to cope with photo-oxidative stress. Here, using *CrAPX4*-knockdown amiRNA lines, the HL-induced oxidative damage and cell death when APX activity was depressed supports that APX is also a key factor in *C*. *reinhardtii* against HL stress. *Arabidopsis* lacking chloroplast APX shows H_2_O_2_ accumulation and oxidized proteins in 1,000 μE·m^−2^·s^−1^ condition^28^. The study of the function of the chloroplast-localized protease, DEG1C, in *C*. *reinhardtii* in HL acclimation responses by Theis et al. (2019)^26^ found that chloroplast CrAPX4 protein showed higher abundance in the *deg1c* mutant compared to the wild type and was involved in HL acclimation. The role of APX in the tolerance to photo-oxidative stress is also reported in plants. The mutants of wheat (*Triticum aestivum* L. cv Sinvalocho MA) that lack TaAPX-6B gene exhibit reduced APX activity appear high susceptibility to HL-induced oxidative stress^29^. So, *C*. *reinhardtii* APX is to scavenge ROS generated under HL condition to acclimate photo-oxidative stress.

The activity of APX is detected in the homogenate of mitochondrium and chloroplast, respectively, but not in the cytosol, while APX activity is higher in the chloroplast than that in the mitochondrium in this green alga (Supplementary Fig. S3). It agrees the subcellular localization of CrAPX isoforms predicted by ChloroP and TargetP that *Chlamydomonas* APX isoforms are localized in the mitochondrium and/or chloroplast (Supplementary Tab. S1). The APX activity in the chloroplast is markedly decreased by 66% in *CrAPX4* knockdown amiRNA lines but the mitochondrial APX activity remains unchanged, reflecting that CrAPX4 contributes to most of chloroplast APX activity in *Chlamydomonas*. This result implies that CrAPX4 is not localized in the mitochondrium. In addition, the proteomic assay of the *Chlamydomonas deg1c* mutant that exhibits a similar protection response to HL stress shows an increase of CrAPX4 protein abundance in the chloroplast stroma^26^. These results evidence that CrAPX4 protein is localized in the chloroplast. Further, the biochemical study shows that the chloroplast APX in *C*. *reinhardtii* is only found in the stroma but not in the thylakoid membrane^30^. However, the thylakoid membrane-associated APX isoforms can be found in the vascular plants^31^. In the future, the organellar localization of APX isoforms in *Chlamydomonas* needs to be identified.

A significant decrease in APX activity in *CrAPX4* knockdown amiRNA lines suggests that CrAPX4 contributes to APX activity. Furthermore, the increase in APX activity due to CrAPX4 expression is crucial for *C*. *reinhardtii* against HL-induced oxidative stress. The downregulation of *CrAPX4* expression results in oxidative damage and cell death under HL condition. Besides, CrAPX4 downregulation does not affect transcript levels of CrAPX1, CrAPX2, and CrVTC2, and both activities and transcript levels of MDAR, DHAR, and GR under NL condition. However, CrAPX4 downregulation results in a change in the expression of enzymes for AsA regeneration under HL condition, in which transcript levels and activities of both CrMDAR1 and CrDHAR1 in *CrAPX4* knockdown amiRNA lines were decreased. Although total AsA content increased in *CrAPX4* knockdown amiRNA lines, their AsA contents show a similar increase to wild type and vector-only lines after 1 h of HL treatment and then appears a less increase after 6-h HL treatment, while DHA content exhibits a further increase as time advanced. Therefore, AsA/DHA ratio appears a less increase in *CrAPX4* knockdown amiRNA lines under HL condition, particularly after prolonged treatment (6 h). Since AsA is a ROS scavenger, a similar increase in AsA content by 1-h HL treatment reflects that the amount of increased AsA efficiently detoxifies ROS and avoid oxidative stress (lipid peroxidation, Fig. 7A) upon a short-term exposure to HL condition. The downregulation of CrAPX4 expression may modulate the signaling pathway leading to a change in AsA hemostasis and regeneration under HL condition. Although AsA pool is enlarged due to enhanced biosynthesis evidenced by increased CrVTC2 transcription under HL condition, the decrease in APX activity together with a decrease in AsA level due to its oxidation by accumulated ROS and the suppression of AsA regeneration due to decreased MDAR and DHAR activities in *CrAPX4*-knockdown amiRNA lines leads to a decrease in ROS scavenging capacity. Thus, in addition to a decrease in APX function, the lower AsA level, the depression of AsA regeneration, and a change in redox state (AsA/DHA) also contribute to the induction of severe oxidative damage and cell death in *CrAPX4*-knockdown amiRNA lines after prolonged HL treatment. Overall, enhanced CrAPX4 expression and its association with sufficient AsA accumulation and the regulation of MDAR and DHAR expression for AsA regeneration are critical for *C*. *reinhardtii* against HL stress.

## Materials and methods

### Algal culture and treatment

The cell wall-deficient *C*. *reinhardtii* P.A. Dangeard strain CC-400 cw15 mt + was purchased from the Chlamydomonas Resource Center (USA). Algal cells were photoheterotrophically cultured in 50 mL of Tris–acetate phosphate medium (TAP)^32^ with a trace element solution in a 125-mL flask (PYREX, Germany). The flask was agitated on an orbital shaking incubator (model OS701, TKS Company, Taipei, Taiwan) (150 rpm) under continuous illumination with fluorescent white light at a normal light intensity of 50 μE·m^−2^·s^−1^ (NL) at 28 °C. The CC-400 strain was used for downregulation transformation. The selected downregulation transformants were tested for HL sensitivity. For HL illumination, algal cells that reached a density of 3 × 10^6^ cells·mL^−1^ after 18 h of incubation under NL conditions were centrifuged at 4,000×*g* for 5 min at 28 °C. Then, the cell pellet was resuspended in fresh TAP medium at a cell density of 3 × 10^6^ cells·mL^−1^, and 10 mL of resuspended algal cells was transferred to a 100-mL beaker (internal diameter: 3.5 cm) for preincubation at 28 °C under NL conditions for 1.5 h in an orbital shaker (model OS701, TKS company) at a speed of 150 rpm. To test *CrAPX4* downregulation in amiRNA lines in response to HL conditions, the algal cells were illuminated at 1,400 μE·m^−2^·s^−1^ intensity to determine the survival of the knockdown transformants. After treatment, the algal cells were sampled by centrifugation at 4,000×*g* for 5 min, and then the pellet was fixed using liquid nitrogen and stored in a − 80 °C freezer until analysis.

### Generation of the *CrAPX4*-knockdown amiRNA line

Artificial microRNA targeting the *CrAPX4* gene was created using the method described by Molnar et al. (2009)^33^. The amiRNA plasmids for *CrAPX4* were designed using the Web MicroRNA Designer platform (WMD2; https://wmd2.weigelworld.org/cgi-bin/mirnatools.pl)^34^. The resulting oligonucleotides N313 APX4-amiRNA1-F 5′ ctagt**AGGGATCAACACACCCACATA**tctcgctgatcggcaccatgggggtggtggtgatcagcgctaTATGAGGGTGTGTTGATCCCTg 3′ and N314 APX4-amiRNA1-R 5′ ctagc**AGGGATCAACACCCTCATA**tagcgctgatcaccaccacccccatggtgccgatcagcgagaTATGTGGGTGTGTTGATCCCTa 3′ (bold letters show amiRNA*, underlined letters show amiRNA sequences), which target the translated region of *CrAPX4*, were obtained. These DNA fragments were annealed and ligated downstream of the PSAD promoter in pChlamiRNA3 (SpeI digested) containing the neomycin phosphotransferase II (nptII) gene as a paromomycin resistance marker (Supplementary Fig. S4). The generated plasmid was transformed into CC400 cells by electroporation as described above. The transformed cells were selected from medium containing 10 μg·mL^−1^ paromomycin, and liquid cultures of the amiRNA strains were supplemented with 10 μg·mL^−1^ paromomycin. Several knockdown strains of *Chlamydomonas CrAPX4* mRNA were generated, and then the APX activity was assayed for primary selection. Five strains that exhibited a significant downregulation of APX activity and *CrAPX4* mRNA levels and one vector-only control were obtained in the present study.

### Enzyme activity determination

APX activity was assayed according to Nakoan and Asada (1981)^35^ with some modifications. Five milliliters of algal culture were collected and centrifuged at 4,000×*g* to collect the cells. The enzyme extract was obtained after extraction of algal cells in 0.25 mL of 0.15 M NaH_2_PO_4_-Na_2_HPO_4_ (pH 7.5) buffer containing 5 mM AsA and 10 mg polyvinylpyrrolidone by sonication. The crude extract was centrifuged at 12,000×*g* at 4 °C for 10 min, and the supernatant was collected as an enzyme extract. For APX activity determination, the enzyme extract was mixed with 0.15 M Na_2_HPO_4_/NaH_2_PO_4_ buffer (pH 7.5) containing 5 mM AsA, 0.75 mM Na_2_EDTA, 10 mM H_2_O_2_, and H_2_O in a total volume of 1 mL at 25 °C with detection at 290 nm. A change in absorbance was detected at 290 nm to estimate activity using an extinction coefficient of 2.8 mM^−1^·cm^−1^.

MDAR activity was assayed according to Yeh et al. (2019)^18^, DHAR activity was assayed according to Lin et al. (2016)^19^, and GR activity was assayed according to Lin et al. (2018)^20^. The soluble protein content was determined according to Bradford (1976)^36^.

### Thiobarbituric acid reacting substance (TBARS) assay

Thiobarbituric acid reactive substances (TBARS) is a standard marker for lipid peroxidation induced oxidative stress^35^. Five milliliters of algal culture were sampled after treatment and centrifuged at 4,000×*g* for 5 min. The pellet was fixed in liquid nitrogen and mixed vigorously with 0.5 mL of 5% (w/v) trichloroacetic acid (TCA). The mixture was subjected to three freezing (− 80 °C)-thaw (25 °C) cycles and centrifuged at 12,000×*g* for 10 min at 4 °C. The supernatant was collected for the determination of lipid peroxidation, and the extent of lipid peroxidation was estimated from the TBARS content determined based on the A_532_–A_600_ with an extinction coefficient of 155 mM^−1^·cm^−1^ and expressed as pmol· (10^6^ cells)^−1^ according to Health and Packer (1968)^37^.

### Cell growth determination

Ten μL of algal culture was mixed with 30 μL of Lugol’s solution (Sigma-Aldrich), and the cell number was counted in duplicate under a light microscope (BX43, Olympus, Tokyo, Japan) using a hemocytometer (Improved Neubauer, Boeco, Germany). The cell number was calculated following the manufacturer’s instructions and expressed as 10^6^ cells·mL^−1^.

### ROS production determination

ROS (H_2_O_2_) were assayed according to our previous study by staining the cells with 2′, 7′-dichlorodihydrofluorescein diacetate (H_2_DCFDA) (Invitrogen, Corporation, USA), which can be digested by cellular esterase to 2′,7′-dichlorofluorescein (DCF)^38^. The production of ROS was determined DCF reacted with ROS (mainly react with H_2_O_2_) with green fluorescence emission. Then, cells fluorescence were observed with a fluorescence spectrophotometer (model F2700, Hitachi, Tokyo, Japan) at excitation wavelength of 492 nm and an emission wavelength of 533 nm.

### Quantitative real-time PCR detection of transcript level

Algal cells of 5 mL were harvested from 5 mL for extraction of total RNA using TriPure Isolation Reagent (Roche Applied Science, Mannheim, Germany) according to the manufacturer’s instructions. The integrity of the RNA was confirmed by visual inspection of the two rRNAs, 18S and 28S, through 1% agarose gel electrophoresis. Then, the extracted total RNA was treated with DNase (TURBO DNA-*free*TM Kit, Ambion, Inc., The RNA Company, USA) and then total DNA-free RNA was used to prepare cDNA according to our previous study^﻿39﻿^. The cDNA was adjusted to a concentration of 30 ng·mL^−1^ of the original RNA amount for quantitative real-time PCR detection of transcript level using a LightCycler 480 SYBR Green I Master Kit (Roche Applied Science, Mannheim, Germany) and the LightCycler 480 Instrument (Roche Applied Science, Mannheim, Germany). The primers for *CrVTC2, CrAPX1*, *CrAPX2*, *CrAPX4, CrMDAR1, CrDHAR1, CrGSHR1, CrGSHR2*, and ubiquitin-conjugating enzyme E2 isoform (*CrUBC*, NCBI: AY062935) were listed in Supplementary Tab. S2. After optimizing the primer and cDNA template concentrations, the primer concentration of 3 μM and the cDNA template concentration of 30 ng·μL^−1^ were used for PCR. The PCR was performed firstly by incubation at 95 °C for 5 min and then 50 amplification cycles including annealing at 60 °C for 10 s, elongation at 72 °C for 5 s, and denaturation at 95 °C for 15 s. The 2^−ΔΔCT^ method based on the CT (cycle threshold) values was used to calculate the relative change in transcript level normalized to the internal control gene, *CrUBC*. Then, the fold increase was estimated in relative to the NL control at 0 h to examine the treatment effect.

### Statistics

Three biological replicates were performed with each beaker as a replicate. All experiments were repeated three times and because similar results were obtained, the results of one experiment are shown. According to our previous study of Lin et al. (2016)^19^, the statistical analyses were first performed by ANOVA test and then significant difference analysis of variance of the control and treatment groups using Duncan’s new multiple range test or Student *t*-test (*P* < 0.05) by SPSS software (SPSS 15.0 for Windows Evaluation Version, Chicago, IL, USA).

## Supplementary information

Supplementary Information.

## Data Availability

All relevant data are included in the manuscript and the Supporting Information files.
