# Supplementary material for: Ascorbate peroxidase 4 plays a role in the tolerance of Chlamydomonas reinhardtii to photo-oxidative stress
Source: Sci Rep. 2020 Aug 6;10:13287. doi: 10.1038/s41598-020-70247-z (PMC7414030; doi:10.1038/s41598-020-70247-z)
Supplement: Supplementary file 1 — Supplementary Information. [file 41598_2020_70247_MOESM1_ESM.pdf]

Supplementary information

Ascorbate peroxidase 4 plays a role in the tolerance of *Chlamydomonas reinhardtii* to photo-oxidative stress

Eva YuHua Kuo[a,b,1], Meng-Siou Cai[a,1], & Tse-Min Lee[a,b,*]

[a]Department of Marine Biotechnology and Resources, National Sun Yat-sen University, Kaohsiung 80424, Taiwan

[b]Doctoral Degree Program in Marine Biotechnology, National Sun Yat-sen University, Kaohsiung 80424, Taiwan

[1]equally distribution

Address correspondence to: Tse-Min Lee, Department of Marine Biotechnology and Resources, National Sun Yat-sen University, Kaohsiung 80424, Taiwan, Tel: +886-7-5252000 ext. 5110, e-mail: tmlee@mail.nsysu.edu.tw

A. pChlamiRNA3 vector nucleotide sequences

```
   1 tatgggtgtt gggtcggtgt ttttggtctt ggttggggtg ttggtggtgc tggtggaaca
  61 tgtcaacatg cccaggaaac caaggcgcgc tagcttcctg ggcgcagtgt tccagctact
 121 agtagccgga acactgccag gaaggagggg gaggctgggt gggagaagcg gtgtggggcg
 181 gattagcctt ggagaccgat tgctttgggt tagtttgggc tggcatagtt tgggctggct
 241 tagttacacc tctagatggc agcagctgga ccgcctgtac catggagaag agctttactt
 301 gccgggatgg ccgatttcgc tgattgatac gggatcggag ctcggaggct ttcgcgctag
 361 gggctaggcg aagggcagtg gtgaccaggg tcggtgtggg gtcggcccac ggtcaattag
 421 ccacaggagg atcaggggga ggtaggcacg tcgacttggt ttgcgacccc gcagttttgg
 481 cggacgtgct gttgtagatg ttagcgtgtg cgtgagccag tggccaacgt gccacaccca
 541 ttgagaagac caaccaactt actggcaata tctgccaatg ccatactgca tgtaatggcc
 601 aggccatgtg agagtttgcc gtgcctgcgc gcgccccggg ggcgcagttt agctgaccag
 661 ccgtgggatg atgcacgcat ttgcaaggac agggtaatca cagcagcaac atggtgggct
 721 taggacagct gtgggtcagt ggacggacgg caggggaggg acggcgcagc tcgggagaca
 781 gggggagaca gcgtgactgt gcaatgcggc cgccaccgcg gtggagctcc aattcgccct
 841 atagtgagtc gtattacgcg cgctcactgg ccgtcgtttt acaacgtcgt gactgggaaa
 901 accctggcgt tacccaactt aatcgccttg cagcacatcc ccctttcgcc agctggcgta
 961 atagcgaaga ggcccgcacc gatcgccctt cccaacagtt gcgcagcctg aatggcgaat
1021 gggacgcgcc ctgtagcggc gcattaagcg cggcgggtgt ggtggttacg cgcagcgtga
1081 ccgctacact tgccagcgcc ctagcgcccg ctcctttcgc tttcttccct tcctttctcg
1141 ccacgttcgc cggctttccc cgtcaagctc taaatcgggg gctcccttta gggttccgat
1201 ttagtgcttt acggcacctc gaccccaaaa aacttgatta gggtgatggt tcacgtagtg
1261 ggccatcgcc ctgatagacg gtttttcgcc ctttgacgtt ggagtccacg ttctttaata
1321 gtggactctt gttccaaact ggaacaacac tcaaccctat ctcggtctat tcttttgatt
1381 tataagggat tttgccgatt tcggcctatt ggttaaaaaa tgagctgatt taacaaaaat
1441 ttaacgcgaa ttttaacaaa atattaacgc ttacaattta ggtggcactt ttcggggaaa
1501 tgtgcgcgga acccctattt gtttattttt ctaaatacat tcaaatatgt atccgctcat
1561 gagacaataa ccctgataaa tgcttcaata atattgaaaa aggaagagta tgagtattca
1621 acatttccgt gtcgccctta ttcccttttt tgcggcattt tgccttcctg tttttgctca
1681 cccagaaacg ctggtgaaag taaaagatgc tgaagatcag ttgggtgcac gagtgggtta
1741 catcgaactg gatctcaaca gcggtaagat ccttgagagt tttcgccccg aagaacgttt
1801 tccaatgatg agcactttta aagttctgct atgtggcgcg gtattatccc gtattgacgc
1861 cgggcaagag caactcggtc gccgcataca ctattctcag aatgacttgg ttgagtactc
1921 accagtcaca gaaaagcatc ttacggatgg catgacagta agagaattat gcagtgctgc
1981 cataaccatg agtgataaca ctgcggccaa cttacttctg acaacgatcg gaggaccgaa
2041 ggagctaacc gcttttttgc acaacatggg ggatcatgta actcgccttg atcgttggga
2101 accggagctg aatgaagcca taccaaacga cgagcgtgac accacgatgc ctgtagcaat
2161 ggcaacaacg ttgcgcaaac tattaactgg cgaactactt actctagctt cccggcaaca
```

2221 attaatagac tggatggagg cggataaagt tgcaggacca cttctgcgct cggcccttcc
2281 ggctggctgg tttattgctg ataaatctgg agccggtgag cgtgggtctc gcggtatcat
2341 tgcagcactg gggccagatg gtaagccctc ccgtatcgta gttatctaca cgacggggag
2401 tcaggcaact atggatgaac gaaatagaca gatcgctgag ataggtgcct cactgattaa
2461 gcattggtaa ctgtcagacc aagtttactc atatatactt tagattgatt taaaacttca
2521 tttttaattt aaaaggatct aggtgaagat cctttttgat aatctcatga ccaaaatccc
2581 ttaacgtgag ttttcgttcc actgagcgtc agaccccgta gaaaagatca aaggatcttc
2641 ttgagatcct ttttttctgc gcgtaatctg ctgcttgcaa acaaaaaaac caccgctacc
2701 agcggtggtt tgtttgccgg atcaagagct accaactctt tttccgaagg taactggctt
2761 cagcagagcg cagataccaa atactgtcct tctagtgtag ccgtagttag gccaccactt
2821 caagaactct gtagcaccgc ctacatacct cgctctgcta atcctgttac cagtggctgc
2881 tgccagtggc gataagtcgt gtcttaccgg gttggactca agacgatagt taccggataa
2941 ggcgcagcgg tcgggctgaa cggggggttc gtgcacacag cccagcttgg agcgaacgac
3001 ctacaccgaa ctgagatacc tacagcgtga gctatgagaa agcgccacgc ttcccgaagg
3061 gagaaaggcg gacaggtatc cggtaagcgg cagggtcgga acaggagagc gcacgaggga
3121 gcttccaggg ggaaacgcct ggtatcttta tagtcctgtc gggtttcgcc acctctgact
3181 tgagcgtcga tttttgtgat gctcgtcagg ggggcggagc ctatggaaaa acgccagcaa
3241 cgcggccttt ttacggttcc tggccttttg ctggcctttt gctcacatgt tctttcctgc
3301 gttatcccct gattctgtgg ataaccgtat taccgccttt gagtgagctg ataccgctcg
3361 ccgcagccga acgaccgagc gcagcgagtc agtgagcgag gaagcggaag agcgcccaat
3421 acgcaaaccg cctctccccg cgcgttggcc gattcattaa tgcagctggc acgacaggtt
3481 tcccgactgg aaagcgggca gtgagcgcaa cgcaattaat gtgagttagc tcactcatta
3541 ggcaccccag gctttacact ttatgcttcc ggctcgtatg ttgtgtggaa ttgtgagcgg
3601 ataacaattt cacacaggaa acagctatga ccatgattac gccaagcgcg caattaaccc
3661 tcactaaagg gaacaaaagc tgggtacccg cttcaaatac gcccagcccg cccatggaga
3721 aagaggccaa aatcaacgga ggatcgttac aaccaacaaa attgcaaaac tcctccgctt
3781 tttacgtgtt gaaaaagact gatcagcacg aaacggggag ctaagctacc gcttcagcac
3841 ttgagagcag tatcttccat ccaccgccgt tcgtcagggg gcaaggctca gatcaacgag
3901 cgcctccatt tacacggagc ggggatccca acgtccacac tgtgctgtca cccacgcgac
3961 gcaaccctac ccagccacca acaccatcag gtccctcaga agaactcgtc caacagccgg
4021 taaaacgcca gcttttcctc cgataccgcc ccatcccacc cgcgcccgta ctcccgcagg
4081 aacgccgcgg aacactccgg cccgaaccac gggtcctcct cgtgggccag ctcgcgcagc
4141 accagcgcga gatcggagtg ccggtccgca cggccgaccc gccccacgtc gatcagcccg
4201 gtcacctcgc aggtacgagg gtcgagcagc acgttgtccg ggcacaggtg accgtggcaa
4261 accgccagat cctcgtccgc aggccgagtc cgctccagct cggcgagaag ccgctccccc
4321 gaccacccct tccgctcctc gtccagatcc tccaagtcga cgctcccttc agcgacagca
4381 cgggccgcct gcggcaccgt caccgcgaga ctgcgatcga acggacaccg ctcccagtcc
4441 agcgcgtgca gcgaacgagc gagccccgcg agcgccaccg ccacgtccag ccgctgctcc

4501 cgcggccacc gcgcactggc cggacgcccc ggaaccgctt cggtgaccaa ccaggcgacc
4561 ctctcgtccc caccaccctc cacaacacga ggtacgggaa tccccacctc cgccaaccac
4621 accagccgct cagcctcacc caacaagccc accccggccc ccagagctgc caccttgaca
4681 aacaactccc gcccaccacc ccgaagccga taaacaccag cccccgaggc cccatcctcc
4741 acaacaaccc actcacaacc gggataccga ccccgcagtg cacgcaacgc atcgtccatg
4801 cttcgaaatt cttcagcacc ggggagggcg gagtggccat cctgcaaatg gaaacggcga
4861 cgcagggtta gatgctgctt gagacagcga cagaggagcc aaaagccttc gtcgacacaa
4921 tgcgggcgtt gcaagtcaaa tctgcaagca cgctgcctga tccgccgggc ttgctcgtcg
4981 actcacctgg ccattttaag atgttgagtg acttctcttg taaaaaagta aagaacatag
5041 gccccctggc cggtttatca ggagggcacc gctccagggg ctgcatgcga actgcttgca
5101 ttggcgccta gcctttgtgg gccagggggc ttccggataa gggttgcaag tgctcaaata
5161 ccccatcaaa catcatcctg gtttggctgc gctccttctg gcgcgcccgg catgcaagct
5221 tgatgggatc ttaagctagc tgagtggtta tgtatagcgg cagaatagtc gcgtatgtat
5281 aagtgctcgt ttgtcgctga aagtggaggt caccgttcgg ggtcgcgggc ttttataccg
5341 gatgggtgcc gccagcgggc cgtatggcgc cttctggacg ccgcgcgccc catcgcggcc
5401 cttccagagc ttcccgcgcc ctcatagccc gccaaatcag tcctgtagct tcatacaaac
5461 atacgcacca atcatgtcaa gcctcagcga gctccccgct cgagcacaca cctgcccgtc
5521 tgcctgacag gaagtgaacg catgtcgagg gaggcctcac caatcgtcac acgagccctc
5581 gtcagaaaca cgtctccgcc acgctctccc tctcacggcc gaccccgcag ccccttttgcc
5641 ctttcctagg ccaccgacag gacccaggcg ctctcagcat gcctcaacaa cccgtactcg
5701 tgccagcggt gcccttgtgc tggtgatcgc ttggaagcgc atgcgaagac gaaggggcgg
5761 agcaggcggc ctggctgttc gaagggctcg ccgccagttc gggtgccttt ctccacgcgc
5821 gcctccacac ctaccgatgc gtgaaggcag gcaaatgctc atgtttgccc gaactcggag
5881 tccttaaaaa gccgcttctt gtcgtcgttc cgagacatgt tagcagatcg cagtgccacc
5941 tttcctgacg cgctcggccc catattcgga cgcaattgtc atttgtagca caattggagc
6001 aaatctggcg aggcagtagg cttttaagtt gcaaggcgag agagcaaagt gggacgcggc
6061 gtgattattg gtatttacgc gacggcccgg cgcgttagcg gcccttcccc caggccaggg
6121 acgattatgt atcaatattg ttgcgttcgg gcactcgtgc gagggctcct gcgggctggg
6181 gagggggatc tgggaattgg aggtacgacc gagatggctt gctcgggggg aggtttcctc
6241 gccgagcaag ccagggttag gtgttgcgct cttgactcgt tgtgcattct aggaccccac
6301 tgctactcac aacaagccca

B. Vector map

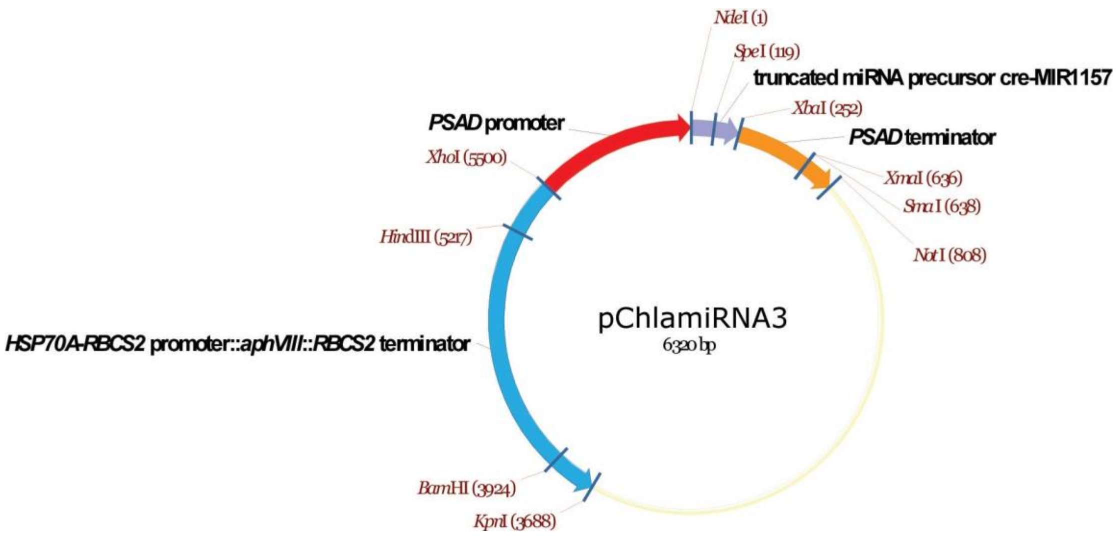

Supplementary Figure S1: The nucleotide sequences (A) and map (B) of pChlamiRNA3 vector.

Supplementary Figure S1A showed the whole nucleotide sequences of pChlamiRNA3 vector in a total bp of 6,320. As shown in Supplementary Figure S1B, the pChlamiRNA3 vector contained PSAD promoter and PSAD terminator. The vector contained the antibiotic resistance to paromomycin with the regulation under the promoter of HSP70A-RBCS2.

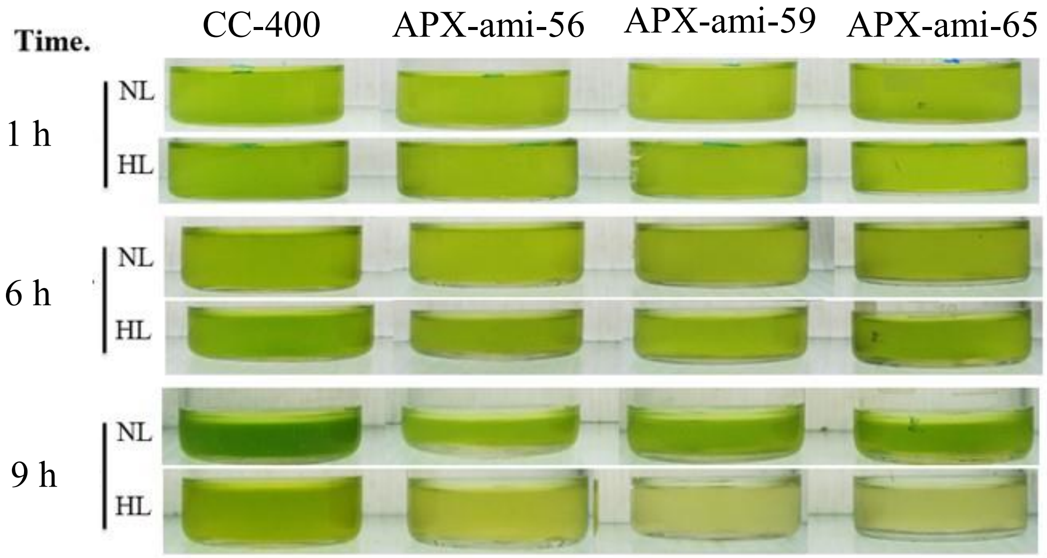

Supplementary Figure S2. The appearance of the *Chlamydomonas reinhardtii* wild type (CC-400) and CrAPX4 downregulation lines after exposure to HL conditions.

NL: 50 μE·m^{-2}·s^{-1}; HL: 1,400 μE·m^{-2}·s^{-1}. The CrAPX4 downregulation lines bleached 9 h after HL treatment while they remained green appearance at 6 h of HL treatment.

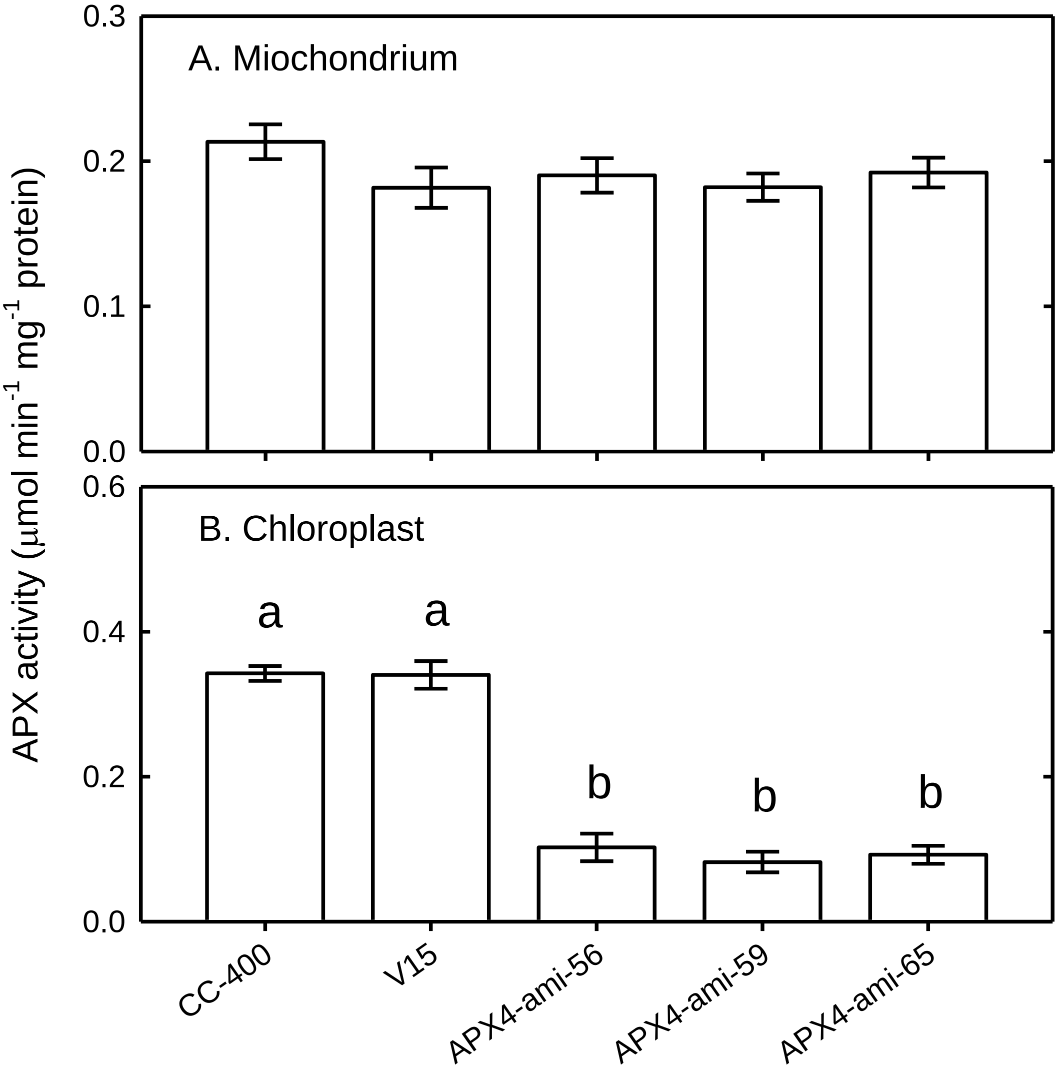

Supplementary Figure S3. APX activity in the mitochondrium (A) and chloroplast (B) of the *Chlamydomonas reinhardtii* cells. The wild type, vector-only line, and CrAPX4 downregulation lines are used.

The activity of APX cannot be detected in the cytosolic faction even increasing the soluble protein amount and the substrate, ascorbate, concentration under different pH conditions (pH 5, 6, 7, 8, and 9), while the activity of APX is higher in the chloroplastic fraction than the mitochondrial fraction.

To examine the subcellular distribution of CrAPX protein, homogenates prepared from freshly harvested cells of the cell wall-deficient *C. reinhardtii* strain CC-400 (wild type), vector-only line, and CrrAPX4 downregulated lines were fractionated by

differential centrifugation to obtain the cytosol, mitochondria and chloroplasts as described in the method of van Lis et al. (2005). Algal cells were harvested by centrifugation at 1,000 ×*g* for 5 min and the pellet was washed twice in 50 mM HEPES buffer (pH 7.2). Then, the pellet was re-suspended in 50 mM HEPES buffer (pH 7.2) at a concentration of 0.5 g algal pellet mL^{-1} with glass beads (425–600 μm, Sigma) in a 50 mL plastic centrifugation tube and vortexed on a bench-top vortex mixer at low speed for 0.5 min twice with an interval of 5 min. Then, the cell homogenate was separated into three parts for the purification of the cytosol fraction, mitochondria and chloroplasts. For the cytosolic fraction, the cell homogenate was centrifuged at 100,000 ×*g* for 30 min at 4ºC and the supernatant was collected as the cytosolic fraction. The mitochondria in the cell homogenate were isolated following the method as described by Eriksson et al. (1995). The homogenate was first centrifuged at 1,500 ×*g* for 10 min and then the supernatant was centrifuged at 10,000 ×*g* for 10 min. Then, the pellet was mixed with 30 mL of 20% Percoll containing 0.25 M sorbitol, 10 mM MOPS-KOH (pH 7.2), l mM EDTA, 0.1% BSA and 0.5% polyvinylpyrrolidone (PVP), and centrifuged at 20,000 ×*g* for 60 min. The bottom 3 ml were diluted with 40 ml of wash buffer (pH 7.2, 10 mM potassium phosphate, 0.1% BSA, 0.25 M sorbitol, 1 mM EDTA) and spun for 10 min at 10,000 ×*g*. The pellet was the isolated mitochondria.

The chloroplasts were isolated from the cell homogenate by centrifugation at 2,000 ×*g*. The pellet was re-suspended in 50 mM HEPES buffer (pH 7.2) containing 0.25 M sorbitol, 10 mM $MgCl_2$, 1 mM $MnCl_2$, 3 mM KH_2PO_4 and 2 mM EDTA. The chloroplasts were recovered at the interface of a 45/75% (v/v) Percoll step gradient in 50 mM HEPES buffer (pH 7.2) containing 0.25 M sorbitol, 10 mM $MgCl_2$, 1 mM $MnCl_2$, 3 mM KH_2PO_4 and 2 mM EDTA after centrifugation at 6,000 ×*g* for 20 min at 4ºC. Isolated chloroplasts were washed three times by sedimenting in the above buffer and then were re-suspended in 10 mM Tricine-NaOH (pH 8.0) in the presence of protease inhibitors (200 mM phenylmethylsulfonyl fluoride, 1 mM benzamidine and 5 mM 6-aminocaproic acid).

Intact mitochondria and chloroplasts were mixed with lysis buffer (50 mM Tris-HCl, pH 7.5) in two cycles of slow freezing to -80ºC followed by fast thawing to room temperature. The soluble cell extract was then separated from the insoluble fraction by centrifugation (15,000 ×*g*, 15 min) at 4ºC and immediately used for activity analysis and protein quantitation. Protein concentrations were quantified with the Coomassie Blue dye binding method (Bradford, 1976) using the concentrated dye purchased from BioRad (catalog number 500-0006; Bio-Rad, Hercules, CA, USA).

References:

Bradford, M. M. A rapid method for the quantitation of microgram quantities of protein utilizing the principle of protein-dye binding. *Anal. Biochem.* **72**, 248-254, https://doi.org/10.1006/abio.1976.9999 (1976).

Eriksson, M., Gardeström, P. & Samuelsson, G. Isolation, purification, and characterization of mitochondria from *Chlamydomonas reinhardtii*. *Plant Physiol.* **107,** 479-483, http://doi.org/10.1104/pp.107.2.479 (1995).

van Lis, R., Atteia, A., Nogaj, L. A. & Beale, S. L. Subcellular localization and light-regulated expression of protoporphyrinogen IX oxidase and ferrochelatase in *Chlamydomonas reinhardtii*. *Plant Physiol.* **139,** 1946-1958, http://doi.org/ 10.1104/pp.105.069732 (2005).

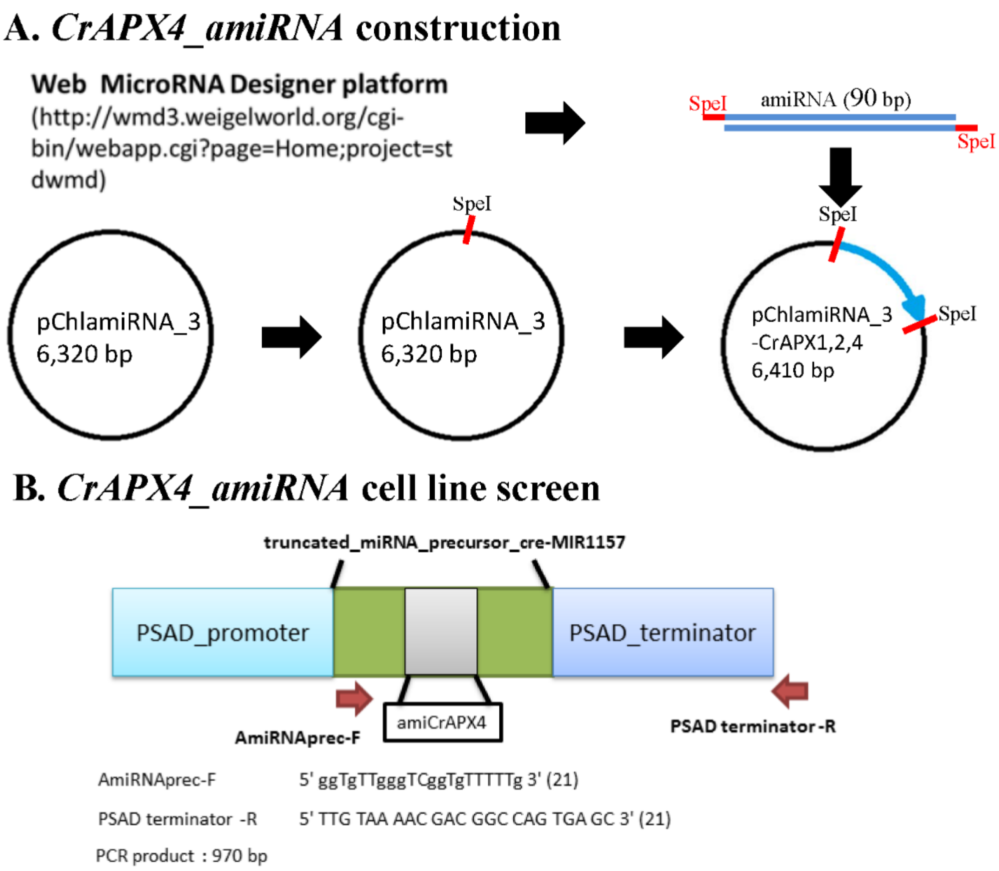

Supplementary Figure S4. Construction of the *Chlamydomonas reinhardtii* CrAPX4 downregulation vector.

The CrAPX4 downregulation plasmid was performed by ligating the DNA fragment designed using the Web MicroRNA Designer platform (WMD2; http://wmd2.weigelworld.org/cgi-bin/mirnatools.pl) in the pChlamiRNA3 vector with Spe1 restriction enzyme cutting sites.

Supplementary Figure S4 illustrated that the method of nuclear transformation by electroporation (Shimogawara et al., 1998) was performed with minor modifications. A linear fragment consists of the CrAPX4 gene (CDS region) and the promoter HSP70A:RBCS2 (Schroda et al., 2000), accompanied by the aphIV gene conferring hygromycin resistance (Berthold et al. 2002) and the β2-tubulin promoter, was gel purified using a Qiagen gel purification kit after digesting the pChamy3 vector with ScaI (New England Biolabs). A 15-mL aliquot of cultured cells in the density of 1.5×10^{6} cells mL^{-1} was harvested by centrifugation at 1,200 ×*g* for 10 min. The algal cell pellets were then suspended in 250 mL TAP medium containing 40 mM sucrose in a concentration of 1×10^{8} cells mL^{-1}. For each transformation, 2 mg of the linear

pChlamy3-CrAPX4 fragment containing the aphIV gene and 50 mg of salmon sperm DNA (Cat#15632-011; Invitrogen) were mixed with 1×10^8 algal cells. Subsequently, the mixture was placed into an electroporation cuvette (a 4 mm gap) (Bio-Rad, Hercules, CA, USA). Using exponential mode, an electric pulse of 600 V and capacitance of 50 mF was applied to the mixture for 11 ms. The algal cells in 125 mL were mixed gently with 5 mL of TAP medium without 40 mM sucrose and cultured in a 6-well cell culture plate for 1 d under 25°C with illumination at 50 μmol m^{-2} s^{-1} intensity and shaking at a speed of 125 r.p.m.). After centrifugation at 1,200 ×*g* for 10 min, the cell pellet was suspended in 200 μL of TAP medium and spread onto a TAP agar plate containing 10 mg mL^{-1} hygromycin. After 5 d of culture for selection of colonies resistant to hygromycin were observed and picked up for culture in 1 mL TAP medium under 25°C with illumination at 50 μmol m^{-2} s^{-1} intensity and shaking at a speed of 125 r.p.m.

Reference:

Shimogawara, K., Fujiwara, S., Grossman, A. & Usuda, H. High efficiency transformation of *Chlamydomonas reinhardtii* by electroporation. Genetics **148**, 1821-1828. https://doi.org/PMC1460073 (1998).

Schroda, M., Blocker, D. & Beck, C. The HSP70A promoter as a tool for the improved expression of transgenes in *Chlamydomonas*. *Plant J.* **21**, 121-131. https://doi.org/10.1046/j.1365-313x.2000.00652.x (2000).

Berthold, P., Schmitt, R. & Mages, W. An engineered Streptomyces hygroscopicus aph 70 gene mediates dominant resistance against hygromycin B in *Chlamydomonas reinhardtii*. *Protist* **153**, 401-412. https://doi.org/10.1078/14344610260450136 (2002).

Supplementary Table S1. Transit peptide analysis of CrAPX isoenzymes by ChloroP 1.1 (http://www.cbs.dtu.dk/services/ChloroP/) and TargetP 1.1 (http://www.cbs.dtu.dk/services/TargetP/).

cTP, chloroplast transit peptides; mTP, mitochondrium transient peptide; CS score, the cleavage site score for each position; RC, reliability class that is a measure of the size of the difference (diff) between the highest (winning) and the second highest output scores.

(A) CrAPX1

	chloroplast	cTP	CS score	cTP length	mTP	other	signal peptide	RC	prediction
ChloroP	0.497	Y	10.042	26					chloroplast
TargetP		0.360			0.745	0.101	0.016	4	mitochondrial

(B) CrAPX2

	chloroplast	cTP	CS score	cTP length	mTP	other	signal peptide	RC	prediction
ChloroP	0.504	Y	3.819	25					chloroplast
TargetP		0.318			0.422	0.113	0.002	5	mitochondrial

(C) CrAPX4

	chloroplast	cTP	CS score	cTP length	mTP	other	signal peptide	RC	prediction
ChloroP	0.559	Y	4.324	28					chloroplast
TargetP		0.698			0.351	0.085	0.005	4	chloroplast

Supplementary Table S2. **The primers for quantitative real-time PCR.**

Primer	Sequence
CrUBC-F	5' CATTAGAGGCGGGCAAA 3'
CrUBC-R	5' TATCGTCATCGTGGTTGTGTAT 3'
CrVTC2-F	5’ GGGTTGGCTTCAAGGTGTGG 3’
CrVTC2-R	5’ TTGTCTCTTGGCCCCGTCTC 3’
CrAPX1-F	5' ACTTTGCGAGCACATTACT 3'
CrAPX1-R	5' GACTCATCAGGCAGGACA 3'
CrAPX2-F	5' CAAGAGGTGAACACGGTAGA 3'
CrAPX2-R	5' AAGATTCCATTTCACATGTCAAC 3'
CrAPX4-F	5' CCTCCCTAAGGCAGCCA 3'
CrAPX4-R	5' GCCACAACTCGCTCAAA 3'
CrMDAR1-F	5' GCTTGGAGCATATTCGAGGG 3'
CrMDAR1-R	5' CACGAACAGCCAGCTCT 3'
CrDHAR1-F	5' ACGGCACAGAGGCCATCATC 3'
CrDHAR1-R	5' CCCCTCCGTAAGCCCTCAAA 3'
CrGSHR1-F	5' GCCATCAAGGTGGATGAGTT 3'
CrGSHR1-R	5' ATAGTCGGGCTTGGTCAGC 3'
CrGSHR2-F	5' TCCTTTCGAGGGAGAGC 3'
CrGSHR2-R	5' GTCATTCTATACACGCCTTCCTA 3'
